# Public health implication of solid waste generated by households in Bekwarra Local Government area

**DOI:** 10.4314/ahs.v21i3.58

**Published:** 2021-09

**Authors:** Donald Ikwun Omang, Godwin Egbe John, Simon Alain Inah, Jude Owan Bisong

**Affiliations:** 1,2 Department of Microbiology, Faculty of Biological Science, University of Calabar, Nigeria; 3 Department of Public Health, Faculty of Allied Medical Science, University of Calabar, Nigeria

**Keywords:** Municipal waste, public health, infection, health hazard, environmental pollution

## Abstract

**Background:**

This study was conducted in Bekwarra Local Government Area of Cross River State, Nigeria, to determine the public health implication of solid waste generated by households.

**Methods:**

A cross sectional descriptive design was employed, using a semi-structured questionnaire together with an observation checklist to elicit information from the respondents. Proportionate sampling was used to select 400 respondents of 18 years and above for the study area. Data collected were analysed using the Microsoft Excel 2007 and Statistical Package for Social Sciences (SPSS) software version 20.

**Results:**

Respondents knowledge concerning solid waste disposal was assessed and the results showed that majority of the respondents 193 (63.7%) had high level of knowledge of solid waste disposal, while 170 (42.5%) had average level of knowledge of solid waste disposal. Wastes produced by households in the study include vegetables (95.5%), ash (94%), clothing/rag (94.2%), wood (95%), and animal waste (86.2%) had the highest abundance. Diseases associated with these wastes produced by households include cholera (18.2%), malaria (47.2%), lassa fever (10.7%) and diarrhea (23.9%) with malaria been the most prevalence infection.

**Conclusion:**

The result shows solid waste posed a serious health hazard and lead to the spread of infectious diseases. These issues can be addressed through health education and enlightenment of the people on waste disposal.

## Introduction

Solid waste comprises all the waste arising from human and animal activities and by-products of processes, which are normally solid, and are discarded as useless or unwanted materials that may be required by law to be disposed off. Solid waste can be classified in a number of ways on the basis of their sources, environmental risks, utility and physical property. Based on origin or source, solid wastes are classified as: Municipal solid waste, Industrial solid waste and Agricultural Solid Waste^1^. The rural communities and urban towns are today struggling to clear heaps of solid waste from their environments. These strategic centers are now being overtaken by overflowing dumps and uncollected heaps of solid waste emanating from household or domestic sources, markets, shopping and business centers^2^. The local government councils are unable to carry out their statutory responsibilities of managing municipal solid in the towns and cities which is a clear violation of the environmental sanitation edicts and regulation. These solid wastes have become threats to the health of the dwellers and it is no longer in doubt that Nigerian cities are overwhelmed with the challenges of uncleared solid waste^3^.

Improper solid waste disposal causes pollution of air, soil, and water while indiscriminate dumping of waste contaminates surface and ground water supplies. In urban areas, solid waste clogs drains, creating stagnant water for insect breeding and floods during the rainy^4^. Uncontrolled burning of solid wastes and improper incineration contributes significantly to urban air pollution. Greenhouse gases are generated from the decomposition of organic wastes in landfills, and untreated leachate pollutes surrounding soil and water bodies. Health and safety issues also arise from improper solid waste management. Insect and rodent vectors are attracted to the waste and can spread diseases such as cholera and dengue fever^5^. Using water polluted by municipal solid wastes for bathing, food irrigation and drinking can also expose individuals to disease organisms and other contaminants^6^. The U.S. Public Health Service identified 22 human diseases that are linked to improper solid waste management. Waste worker and pickers in developing countries are seldom protected from direct contact and injury^7^. Exhaust fumes from waste collection vehicles, dust stemming from disposal practices and the open burning of waste also contribute to overall health problems^8^.

Elizabeth et al.^9^ reported that indiscriminate waste dumping practice caused the increasing incidence of diarrhea among under age children in Odukpani, Akamkpa and Biase Local Government Area of Cross River State, Nigeria. Despite significant investments in the waste management sector, solid waste management remains one of the major environmental sanitation challenges facing the country today and has continually remained at the lowest level, because industrialization and rapid population growth in many cities and towns have led to wastes being generated faster than they are collected, and disposed. The major issues affecting Bekwarra Local Government Area of Cross River State, Nigeria include the absence of proper solid waste collection services, growing number of uncontrolled heaps of rubbish in the communities constituting hazards to public health. In Cross River State, for instance, studies have shown that the Calabar Urban Development Authority (CUDA) is saddled with the responsibility of refuse collection and evacuation but the situation is different in the rural areas, including Bekwarra Local Governmen^10^. Poor public attitude resulting in indiscriminate waste dumps in street corners, in the drains increase the rate of pest and vector breeding vectors and pollution of the environment, air, water and soil. According to Bassey et al.^11^, the disposal of solid waste in the Ikot Effanga Area of Cross River State has caused the pollution of underground water leading to high mortality rate. Therefore, it is in this context that the present study was carried out to assess the level of environmental pollution and the potential impacts on public health in relation to solid waste management in Bekwarra Local Government Area of Cross River State, Nigeria and proffer solutions to them which would help change the negative attitude of the people and lead to improved waste management practices as well as ensure clean environment.

## Methods

### Study Setting

Bekwarra Local Government Area is located in the Northern Senatorial district of Cross River State of Nigeria and was created out of the former Ogoja Local Government Area on 1^st^ October 1996. The Local Government is bounded on the north by Vandekya Local Government of Benue State, on the South by Ogoja Local Government, on the east by Obudu and west by Yala. The Local Government has a projected population of 105,822 and Area of 306km2 (118sqmi) with ten political wards^12^. Most inhabitants of the area are traders, rural farmers and fisherman. Majority of the populace are Christians with few Muslims and traditional religious groups

### Study Design

The design for this study was a cross sectional descriptive study using a self-developed questionnaires and an observation checklist to gather relevant information on solid waste disposal practices in Bekwarra Local Government Area of Cross River State.

### Study Population

The study population included all adult females and males of 18 years and above living in the study area.

### Sample Size Determination

The sample size for this study was determined by using Cochran's formular^13^.



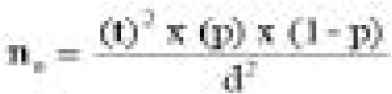



Where:

t= value of selected alpha level of 0.025 (e.g. 1.96 for 95% confidence level)

p = proportion of solid waste disposal= 0.7(70% being the prevalence for Calabar)13

q = (1 - p) = 0.3

d=acceptable margin of error for proportion being estimated = 0.05

no = (1.96)2 x (0.7) (0.3) = 322

(0.05)2

To account for the possible attrition and non-response, the sample size was increased by 25% giving a sample size of 400 which was used as the actual sample size for the study.

### Sampling Procedure

Multi-stage sampling technique was adopted in the selection of study participant and are describes below:

**Stage 1:** Selection of Wards: Out of the ten wards in Bekwarra Local Government Area, five wards were selected using simple random technique (balloting). This was done by writing the names of all the council wards in the Local Government in a sheet of paper and folded. From the folded papers only five (5) were picked without replacement representing five wards for the study after mixing and shuffling in a basket.

**Stage 2:** Selection of Houses and Households: In each selected ward, the total number of houses was obtained from the Primary Health Care Department from house numbering used by the Primary Health Care Department for immunization. Conservative recruitment sampling of households starting from the village square, the chief house respectively was then applied to select the required number of houses/households from the wards.

**Stage 3:** Selection of Respondents: In each household, an adult female/male was selected. In households with many adult females and males, simple random technique (balloting) was carried out. Yes and No were written in a piece of paper and folded. Thereafter, these were placed in a basket and shaken to mix thoroughly, then the adult females/males were asked to pick, the one with the yes was selected for the interview.

### Data Collection

Data was collected from respondents at the household level using the semi-structured questionnaire. The questionnaire comprised of section A - socio-demographic data while section B comprised types of waste generated, methods of waste collection and disposal and self-reported health problems associated with solid waste disposal by respondents. The questionnaire was pre-tested among 5% of the sample population in Yala Local Government Area having similar characteristics with the study area. This was to ensure that the questions were appropriate for the study. Three research assistants with tertiary education were recruited and trained for one week to assist in data collection by the research coordinator. The questionnaire was administered to each respondent after seeking her verbal consent. Proper explanations were given by the researcher when required, and it took about 8 – 15 minutes to administer the questionnaire to each respondent. In all, a period of four (4) weeks was used, from June 2016 to July 2016 to complete the study. The observational check list was also used by the researcher to provide additional information such as services and conveniences, ventilation, waste disposal facility and refuse collection on premises that was not captured in the questionnaire.

### Methods of Data Analysis

Data was analysed using the Microsoft Excel 2007 and Statistical Package for Social Sciences (SPSS) software version 20. The association between variables was tested using the Chi-square. Scores were assigned to each response accordingly and later summed up to get the total score for each individual. Score range 0 – 3 represented low knowledge of waste disposal, score range 4 – 7 represented average knowledge of waste disposal while score range of 8 – 10 represented high knowledge of waste disposal. The minimum score was 0 while the maximum score was 10 out of a possible total of 10.

### Ethical consideration

A letter of introduction was obtained from the Department of Public Health, University of Calabar, to enable the researcher obtain ethical clearance from the Cross River State Research Ethics Committee, Ministry of Health to facilitate access in the Community. Verbal consent was sought from the community heads in Bekwarra Local Government Area where the research was carried out. Informed verbal consent was also obtained from the study participants. The Participants were informed that participation in the study was voluntary and assured of anonymity of their identity before commencement of the survey. In addition, they were also told that at any point in time they could withdraw from the study without consequences.

## Results

**Figure 1:**
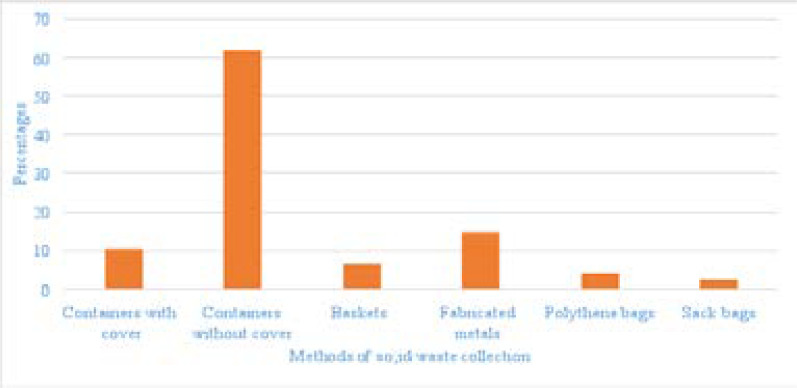
Methods of solid waste collection by household in Bekwarra

**Figure 2:**
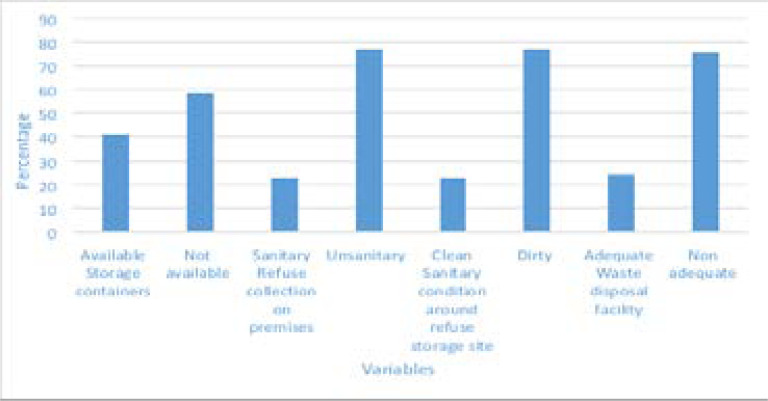
Observation checklist of solid waste disposal method

A total of 400 participants completed and returned the questionnaires giving a response rate of 100% as presented in Table 1. Respondents between the ages 20 – 29 years accounted for majority of the participants 145 (40.6%) while the least respondents were within the age bracket 50 – 59 years of age (9%). Majority of the respondents (49.5%) were engaged in small scale businesses with 27.8% been farmers. Most of the respondents (42.2%) were observed to have secondary education, with only 9.3% that had no formal education. Majority of the respondents (73.4%) of the respondents earned below N10,000, as basic monthly income. The interval in which the respondents reportedly earned their income varied as 47.2% of respondents earned their income daily while 22% earned their income monthly. Majority of the respondents (96.9%) were Christians, married respondents were 62%, while respondents with small family size of less than four children were 43.9%. Among the respondents sampled as shown in Figure 1, majority of the respondents (61.8%) disposed their wastes in containers without cover while only 2.5% of the respondents dump their wastes in sack bags. Results from the observation checklist as presented on Figure 2 showed majority of the storage sites were dirty with no storage containers and inadequate storage facility. Results obtain in Table 2 showed the perceived association between health problems and the method of solid waste disposal.

**Table 1:** Socio Demographic Characteristics of Household Respondents

Variables		Frequency	Percent (%)
	Less than 20	25	7
	20–29	145	40.6
	30–39	85	23.8
Age	40–49	50	14
	50–59	32	9
	60 and above	20	5.6
	**Total**	**357**	**100**
	Farmer	111	27.8
	Small scale business	198	49.5
Occupation	Large scale business	7	1.8
	Civil/public servant	30	7.5
	Student	26	6.5
	Unemployed	28	7
	**Total**	**400**	**100**
	Christianity	373	96.9
	Islam	1	0.3
Religion	Traditional religion	11	2.8
	**Total**	**385**	**100**
	Less than 4 children	145	43.9
Number of	4–6 children	133	40.3
children in family	7 and above children	52	15.8
	**Total**	**330**	**100**
	No formal education	37	9.3
Highest	Primary	81	20.5
educational status	Secondary	167	42.2
	Tertiary	56	14.1
	Dropout	55	13.9
	**Total**	**396**	**100**
	Below N10,000	207	73.4
	N10,000–N19,999	35	12.4
Monthly income	N20,000–N49,999	19	6.7
	N50,000–N99,999	15	5.3
	Above N100,000	6	2.1
	**Total**	**282**	**100**
	Married	246	62
	Single	53	13.4
Marital status	Separated	21	5.3
	Widowed	13	3.3
	Co-habiting	64	16.1
	Total	397	100

**Table 2:** The association between perceived health problems and the method of solid waste disposal

Diseases associated with waste		Waste disposal method					Chi-square	P-value

		Composting	Direct dumping	Bush around	Community dumpsites	Burning		
Malaria	Yes	15(9.6%)	51(32.7%)	22(14.1%)	11(7.1%)	57(36.5%)		
	No	22(12.6%)	39(22.3%)	29(16.6%)	9(5.1%)	76(43.4%)	5.728	0.22
Diarrhea	Yes	12(15%)	11(13.8%)	11(13.8%)	5(6.2%)	41(51.2%)		
	No	25(10%)	79(31.5%)	40(15.9%)	15(6%)	92(36.7%)	11.80	0.019
Cholera	Yes	6(10%)	14(23.3%)	11(18.3%)	4(6.7%)	25(41.7%)		
	No	31(11.4%)	76(28%)	40(14.8%)	16(5.9%)	108(39.9%)	0.987	0.912
Lassa fever	Yes	4(11.4%)	14(40%)	7(20%)	0	10(28.6%)		
	No	33(11.1%)	76(25.7%)	44(14.9%)	20(6.8%)	123(41.6%)	6.578	0.160

*Significant at p < 0.05

The results showed that open dumping contributed to about 51% of malaria.

## Discussion

The study revealed that the composition, storage and disposal of solid waste in Bekwarra Local Government area potentially have environmental and public health implications. Health risk assessment of solid waste management in this area shows that, only about 30% households have appropriate containers as dustbins. The results revealed that government do not provide dustbin to about 30% of the populace, hence the high increase of indiscriminate dumping of municipal solid wastes.

This situation is not different in most local governments in Cross River State including Odukpani, Akamkpa and Biase Local Government Area as reported by Elizabeth et al^9^. It has been noted that improper solid waste disposal and management can lead to air, soil, and water pollution because as it clogs drains, creating stagnant water for insect breeding and transmission of diseases such as cholera. The U.S. Public Health Service identified diseases such as cholera, malaria, dengue fever, respiratory infection, and asthmas as the major health problems associated with improper municipal solid waste management^14^,^15^,^4^. These findings indicate that the air environment around the waste dumpsite is usually contaminated by particulate substances. Although the physicochemical parameters in air were not measured, dust, oxides of nitrogen, sulfur dioxide, carbon monoxide, hydrocarbons, radioactive substances and a trace of toxic elements have been reported to be associated with air pollution caused by solid waste incineration^16^,^11^. The present study showed that 25.3% of the respondents practiced open dumping as a means of waste disposal. This is lower than the figure reported in a study conducted in Abeokuta, whereby 57.9% of the respondents reported that they disposed their wastes in open dump sites to be collected later, probably by a garbage truck^17^. The perception of the respondents in this research is in support of the findings of the research conducted by Aibor and Olorunda^18^ that most diseases are caused by environmental contamination through waste, though not scientifically proven.

From the environmental point of view, the pollution from open dumping of waste could result in the production of not only unsightliness but also in the production of bad odours resulting from the decomposition of solid wastes. From public health point of view, such improper waste disposal techniques can become a serious health hazard through creating a suitable environment from which diseases can be transmitted. A report by PAI Associate International opines that such communicable diseases may be fly borne. They include typhoid, dysentery, cholera, onchocerciasis, sandfly fever and conjunctivitis and many others.

Similarly, a study conducted in Lagos by Adetokunbo and Herbert^5^ revealed that majority of the households used the communal system of waste collection (45%) or throwing waste into the bush (12.5%) as a means of waste disposal. From the public health point of view, improper solid waste management often attracts insect and rodent vectors which facilitate the spread of diseases such as cholera and dengue fever6. Thus, it corroborates the present study which identified cholera 61 (18.2%), malaria 158 (47.2%), lassa fever 36 (10.7%) and diarrhea 80 (23.9%) as diseases associated with solid waste disposal in the study area.

Moreover, central depots where each household can deposit wastes for collection by garbage trucks and tippers are not evenly located along most streets and, in most cases, located very far away from most available space^8^, a situation that is attributed to the high cost of purchasing the dustbins. This situation is not different in most Nigerian cities including Abuja till date, as reported by Aliyu^19^.

## Conclusion

The study focused on the on impact of solid waste on the health conditions of indigenes of Bekwarra Local Government Area of Cross River State, Nigeria due to non-engineering and non-scientific disposal. It is found that with increase in the global population and the rising demand for food and other essentials, there has been a rise in the amount of waste being generated daily by each household. This poses serious health risk and destruction of biodiversity in both terrestrial and aquatic ecosystems of the area. The situation could be aggravated by the poor storage systems in homes and establishments thus constituting good channels for disease transmission by flies, mosquitoes and rats. Further assessments could be needed during the rainy season to have a comparative idea of the level solid waste dumpsite in Bekwarra Local Government Area of Cross River State, Nigeria is of great public health concerns.
